# Linking Vertebrate Gene Duplications to the New Head Hypothesis

**DOI:** 10.3390/biology12091213

**Published:** 2023-09-06

**Authors:** Lindsey Ray, Daniel Medeiros

**Affiliations:** Department of Ecology and Evolutionary Biology, University of Colorado, Boulder, CO 80309, USA

**Keywords:** gene regulatory network, neural crest cells, genome duplication, vertebrates, cyclostomes, new head, pharyngeal arches

## Abstract

**Simple Summary:**

Neural crest cells are unique to vertebrates and migrate long distances throughout the embryo and give rise to many cell and tissue types, including bone, cartilage, smooth muscle, and peripheral nerves. Neural crest cells express many genes that are also expressed in the neural plate border, cartilage, and neurons of invertebrate chordates. However, in neural crest cells, these genes interact as a novel gene regulatory network unique to vertebrates. Much of the vertebrate head is built from neural crest cells, and the evolutionary flexibility of the vertebrate head is thought to have facilitated the evolution of new vertebrate groups. The genomes of vertebrates also show evidence of repeated genome duplication events. However, it is not clear whether these duplications were necessary for the evolution of neural crest cells and thus the vertebrate head. Here, we discuss the general architecture of the gene regulatory network driving neural crest development and highlight gene families within the network that have diverged following duplication events. Based on these analyses, we conclude that the origin of neural crest cells and the neural crest gene regulatory network were not dependent on the vertebrate genome duplications. However, these duplications may have facilitated the diversification of neural crest derivatives, including the head skeleton.

**Abstract:**

Vertebrates have diverse morphologies and various anatomical novelties that set them apart from their closest invertebrate relatives. A conspicuous head housing a large brain, paired sense organs, and protected by a skeleton of cartilage and bone is unique to vertebrates and is a defining feature of this taxon. Gans and Northcutt (1980s) proposed that the evolution of this “new head” was dependent on two key developmental innovations: neural crest cells (NCCs) and ectodermal placodes. NCCs are migratory embryonic cells that form bone, cartilage, and neurons in the new head. Based on genome size, Ohno (1970s) proposed a separate hypothesis, stating that vertebrate genome content was quadrupled via two rounds (2R) of whole genome duplications (WGDs), and the surplus of genetic material potentiated vertebrate morphological diversification. While both hypotheses offer explanations for vertebrate success, it is unclear if, and how, the “new head” and “2R” hypotheses are linked. Here, we consider both hypotheses and evaluate the experimental evidence connecting the two. Overall, evidence suggests that while the origin of the NC GRN predates the vertebrate WGDs, these genomic events may have potentiated the evolution of distinct genetic subnetworks in different neural crest subpopulations. We describe the general composition of the NC GRN and posit that its increased developmental modularity facilitated the independent evolution of NC derivatives and the diversification of the vertebrate head skeleton. Lastly, we discuss experimental strategies needed to test whether gene duplications drove the diversification of neural crest derivatives and the “new head”.

## 1. Introduction

Vertebrates are the most abundant lineage of deuterostomes, comprising about 83% of the species described in the clade [1]. Compared to their invertebrate relatives, vertebrates have elaborated upon the chordate body plan with a range of new cell types tissues, organs, and structures, contributing to more complex morphologies [2,3,4]. Neural crest cells (NCCs) are an embryonic cell type that are unique to vertebrates, which emerge from the neural plate border. During neurulation, NCCs migrate throughout the body to give rise to a diverse array of neural and non-neural cell types including cartilage, bone, smooth muscle, peripheral neurons, and melanocytes [2,3,4,5]. Research on invertebrate chordates has highlighted cells that share some traits with NCCs, including the ability to migrate and give rise to neural or mesenchymal tissues, suggesting that the last common ancestor of chordates and vertebrates had NCC-like cells [2,4,6,7]. However, these invertebrate cell types lack the pluripotency, long-range migratory ability, and spatial awareness of true NCCs [2,7]. The evolution of NCCs was thus a taxon-defining change in development and is thought to have potentiated the species diversity seen in vertebrates. 

The development of NCCs and their derivatives have been studied for over a century, and new technologies are allowing scientists to test the theories about their evolution. The dominant model posits that NCCs evolved by co-opting pre-existing genes and genetic subcircuits from other germ layers, building the neural crest gene regulatory network (NC GRN) in a stepwise fashion [2,3,5,7]. Developmental gene regulatory networks consist of transcription factors that bind to *cis-*regulatory elements to activate or suppress downstream genes, signaling molecules that mediate intercellular communication, and effector genes that determine cellular phenotype. The NC GRN is novel to vertebrates, and understanding how these gene interactions were established will shed light on how macroevolutionary novelties, like new cell types, arise.

The vertebrate head is comprised largely of NCC derivatives and is an evolutionary innovation thought to have facilitated the species richness of this taxon relative to invertebrate chordates [2,4,8]. Gans and Northcutt originally hypothesized the “new head”, as an elaboration of the pre-existing pharyngeal skeleton of chordates, was made possible by NCCs and their migratory, multipotent nature [8]. They speculated that with a muscular and fortified head skeleton, vertebrates were able to evolve forms of active predation and fill many ecological niches, permitting their immense speciation [8]. The new head hypothesis has inspired investigations into the genetic basis of NCC evolution using a variety of developmental and genetic techniques to elucidate conserved and divergent aspects of the vertebrate NC GRN [5,9,10,11].

**Figure 1:**
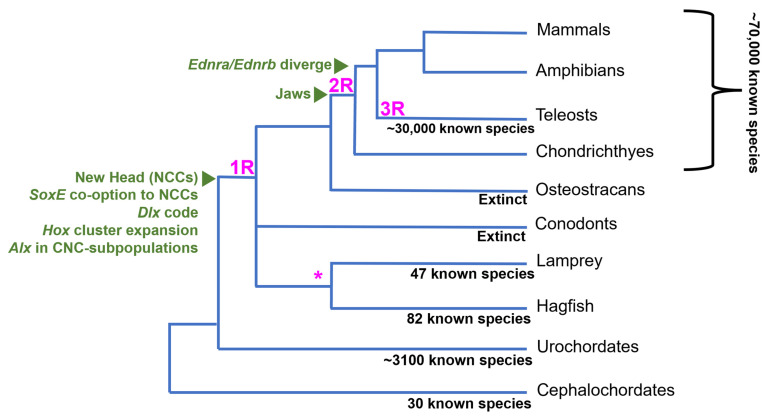
Simple chordate phylogeny—WGDs are shown in pink. A (*) near the cyclostomes indicates possible lineage-specific duplication events of an unknown scale. Adapted from [2,15,16]. Species data from [1].

Another interesting trait separating vertebrates from other chordates is their genome structure and content. The “2R” hypothesis posits that vertebrates quadrupled their genetic material, reducing genetic pleiotropy and permitting mutations to persist without disturbing crucial gene functions [12]. Comparative genomic studies highlight the expansion of the vertebrate genome. Ancestral chordate linkage groups correspond to multiple homologous regions in the vertebrate genomes, indicating a four-fold increase in gene content in vertebrates [13]. Additionally, a third whole genome duplication (WGD) event occurred at the base of teleosts (Figure 1), which are the most species-rich lineage of vertebrates [14]. Taken together, the increase in species diversity following 2R and 3R gene duplication events in vertebrates indicates a positive correlation between genetic material and species diversity, though it is still debated whether WGDs were necessary for the evolutionary expansion of vertebrates.

The fossil record provides some support for the new head hypothesis. While most living species of vertebrates have jaws, there was a large radiation of jawless vertebrates during the Silurian through the end of the Devonian period (~300–400 Ma) [15,17]. Muscularization of the pharyngeal basket and extension of the upper lip allowed jawless stem gnathostomes, like osteostracans, to have powerful suction power and bony cranial structures that allowed them to efficiently capture and crush prey [15]. Prior to this innovation, the chordate pharyngeal skeleton facilitated passive respiration and filter feeding with ciliated structures [18]. Further diversification of vertebrates occurred after the evolution of the jaw, as gnathostomes largely replaced agnathans in the late Devonian [15,17,19]. One study shows there was an increase in active predation after the appearance of jawed fish, indicated by bite marks on fossilized prey or finding fish within the stomachs of fossilized predatory fish [17]. This evidence supports the idea that after the evolution of the new head, jaw evolution further facilitated vertebrate species richness by increasing the predatory capability of jawed fish and allowing them to fill new ecological niches.

The absence of genetic data from extinct, stem vertebrate species makes it difficult to directly link WGDs to vertebrate innovations, like the new head or paired fins. The current estimate of the timing of the WGDs is based on comparative studies between the genomes of invertebrate deuterostomes, cyclostomes, and crown gnathostomes [20,21,22,23]. Recent genomic analyses support at least one WGD at the base of vertebrates, with a second phase of genome-scale duplication occurring in jawed vertebrates [24]. The fossil record also shows evidence for a teleost-specific third WGD (3R) around 50–100 million years ago, as stem teleosts have cell sizes similar to modern species [14]. This suggests a link between WGDs and the evolution of key teleost morphological innovations. However, the large delay between the 3R and teleost radiation suggests it was not a direct driver of teleost diversification [14]. 

It has been proposed that the 2R WGDs may have facilitated the formation of the NC GRN by permitting new gene interactions while maintaining those crucial for development [3,5,22,25]. Many homologs active within the neural plate border (NBP) and underlying mesoderm of invertebrate chordates are active in the vertebrate NBP and neural crest cells that originate from it [2,4,7]. This suggests that ancient genes were co-opted into the NC-GRN from the NPB and other non-ectodermal tissues. It has been proposed that WGDs affect the regulatory landscape of genes more than the functionality of proteins, potentially facilitating gene co-option and GRN evolution [7,20]. Alternatively, elaboration of a pre-existing proto-NC GRN, active in the NPB, or blastula-stage cells, may have occurred before the WGD [25].

While the 2R and new head hypotheses are both used to explain the success of the vertebrate subphylum, these ideas remain largely unlinked [22,23]. This is likely because these hypotheses have very different foundations: the new head hypothesis focuses on developmental and morphological novelties, while 2R is based on genome size and structure. This review will cover the current understanding of the genetic basis of neural crest cell development and evolution and discuss current evidence linking WGDs to the origin and evolution of the NC GRN. In addition, both current and future research efforts to better understand the genetic processes that facilitated the evolution of the NC GRN will be considered. Taken together, evidence suggests that the origin of the NC GRN and neural crest cells occurred before the vertebrate WGDs. However, once in place, the WGDs may have facilitated the evolution of new, independent gene regulatory subcircuits in different NC subpopulations. These new subpopulation-specific NC GRNs may have allowed subpopulations of NC cells, and their derivatives, to evolve independently, contributing to the morphological diversification of the vertebrate new head.

## 2. The NC GRN

### 2.1. Neural Crest Establishment and Migration

During the development of the central nervous system, all chordates deploy a suite of genes to separate neural and non-neural ectoderm, including homologs of *Tfap2*, *Zic*, *Msx1*/*2*, *Pax3*/*7*, and *Dlx3*/*5* [2,5,26,27]. In vertebrates, these genes activate a suite of neural crest marker genes, including *Snail*, *Id*, *Tfap2*, *Twist*, *FoxD3*, and *SoxE* within neural plate border (NPB) cells [2,5]. These NCC specifier genes consist of transcription factors that are deployed at different stages of NCC development. In invertebrate chordates, homologs of most NCC specifiers, with the exception of snails, are absent from the neural border and expressed in other germ layers or ectodermal domains, suggesting they were co-opted to neural border cells in vertebrates [28,29]. As neurulation proceeds, the vertebrate NPB cells expressing a combination of NCC specifier genes become true neural crests.

There are many subnetworks (also known as subcircuits) within the NC GRN that regulate different aspects of the NCC phenotype during different phases of their development including pluripotency, migratory capabilities, and differentiation capacity. These subcircuits use many of the same transcription factors and signaling molecules as the core NC GRN at different times to attenuate the activation and suppression of downstream target genes. NCC marker genes regulate downstream genes that initiate their delamination from the ectoderm during the epithelial-to-mesenchymal transition (EMT), which is marked by the expression of *Snail*, *FoxD3*, *Twist*, *Lmo4*, and *Zeb2* [27]. Regulation of the EMT subcircuit leads to the dynamic expression of cadherin proteins regulated by transcription factors, such as *Snail* and *Zeb2* [30]. Some vertebrates have slight differences in cadherin protein activity during the EMT, but many express type I cadherins prior to delamination and switch to type II cadherins prior to and during migration, orchestrating changes in the cytoskeleton that allow the proper NC movement [27,30,31]. These genes, along with *Ets1*, *c-Myc*, *Tfap2*, *Id*, and *SoxE*, are expressed after NCCs separate from NPB and remain active during their migration throughout the body [5,27]. It is important to note there are differences between the gnathostome and cyclostome NC GRN, as pre-and post-migratory 22NCCs express various NC-specific components at different times and with different expression boundaries, but gene swap experiments show that these homologous proteins are functionally similar between lineages [2,9,27,28]. The evolutionary significance of the heterochronic expression of these components of the NC GRN between vertebrates remains unknown. 

The initial positioning of NCCs along the anterior–posterior axis affects their ultimate post-migratory destination and fate. In gnathostomes, NCCs form four major subpopulations: cranial, vagal, cardiac, and trunk/sacral, all of which give rise to different structures throughout the body [28,32,33]. In the other major lineage of extant vertebrates, the jawless cyclostomes, the precise boundaries, and derivatives of non-cranial NCC subpopulations are less clear. Thus, for the purposes of this review, we will refer mainly to cranial and trunk neural crests. Cranial NCCs migrate into the pharyngeal arches (PAs) in a conserved pattern across vertebrates, where they receive various signals dependent on their anterior–posterior position that determine their skeletal fate after migration [16,28,34]. At the NPB, amniote cranial NCCs (CNCCs) are marked by the co-expression of *Bm3*, *Lhx5*, and *Dmbx1* that activate *SoxE*, *Tfap2*, and *Est1*, which is maintained throughout the migration to the PAs [10]. Recent work in mice revealed that CNCCs reactivate pluripotency marker *Oct4* after delamination and reset their positional information and become transcriptionally equivalent prior to migration into the PAs [35]. Whether this is a conserved feature of the NC GRN, or unique to amniotes, is unclear. 

Skates, a representative of jawed cartilaginous fish, lack the early CNCC markers but deploy *SoxE*, *Tfap2*, and *Ets1* prior to and during migration, while zebrafish share a majority of their CNCC specification and migration GRN with amniotes, with the exception of *bm3* [10]. This evidence highlights a stepwise acquisition of the CNCC GRN in gnathostomes as well as a high conservation of their expression patterns. Lamprey lack the vagal stream of NCCs, with trunk NC-derived Schwan precursor cells giving rise to their enteric nervous system, which stems from vagal NCCs in gnathostomes [28,36,37]. Generally, lamprey CNCCs exhibit a GRN more comparable to a trunk neural crest than those in gnathostomes, with migratory CNCCs being marked with *SoxE* and *Tfap2* [9,10,38]. They also express some genes orthologous to amniote early cranial specifiers, *Lhx5* and *Dmbx1*, later in the PAs [10]. Additionally, the migration patterns of CNCCs in lamprey are less restricted, with the cells destined for the posterior pharyngeal arches migrating initially as a sheet rather than distinct streams [34]. This may be a result of heterochronies between the NCC migratory GRN of jawed and jawless vertebrates [34]. 

### 2.2. Neural Crest Derivatives in the New Head

The vertebrate head skeleton can be divided into the neurocranium, which encases the brain and can have a mesodermal component, and the viscerocranium, which develops from NCCs in the pharyngeal arches [39]. NCCs populate the PAs during head development and give rise to unique structures in each arch. While there are many cranial NCC derivatives that are shared by all vertebrates, hagfish and lamprey lack some NCC derivatives, such as jaws and cranial sympathetic ganglia [34,37]. Due to the inaccessibility of hagfish embryos, the vast majority of what is known about NCC development in agnathans is from studies of lamprey embryos. 

Intercellular signals that are received by CNCCs as they migrate into the head are generally conserved between jawed and jawless vertebrates [16]. Within the PAs, both cyclostomes and gnathostomes develop NC-derived skeletal structures. However, the cartilage composition differs in the pharyngeal skeleton, and lampreys totally lack bone [40]. *Fibroblast growth factor* (*Fgf*) expression is activated by retinoic acid (RA) signaling within the pharyngeal pouch endoderm and is crucial for cartilage development in all vertebrates. FGF receptors on CNCCs receive that signal and activate a downstream cartilage regulatory module containing *Dlx*, *SoxE*, *Twist*, and *Ets* [16,40]. A similar GRN is deployed in the cartilage of amphioxus oral cirri, implying an ancient cartilage regulatory subcircuit may have been co-opted into CNCCs during vertebrate evolution [4]. Chondrocytes differentiate and give rise to cellular and “soft” cartilages that permit the stability and flexibility of the viscerocranium. In addition to the cartilage differentiation genes mentioned above, gnathostomes require *Barx* and *Runx* for proper facial cartilage/bone development, while lamprey only deploy those genes in the branchial vasculature of the PAs [41]. 

All vertebrates deploy a conserved subset of transcription factors within CNCCs, filling the PAs that give rise to different structures and skeletal fates [16]. Within the head, CNCCs give rise to both dermal and endochondral bone [42] Dermal bones, such as mandibular bones forming from the first PA, ossify directly from mesenchymal cell matrices. Endochondral bone, such as the CNC-derived parietal bone of the skull, requires a cartilage intermediate prior to ossification [42]. Just before FGF signaling activates chondrogenesis, other signaling pathways including BMP, endothelin (Edn), and *Notch* initiate the patterning of the CNC and resulting head [16,23]. *Twist*, *Ets*, *Id*, *Alx*, and *SoxE* orthologs are expressed in all vertebrate skeletogenic CNCCs during migration and the population of the PAs [41]. Skeletal elements differentiate through the polarized and combinatorial expression of *Alx*, *Hand*, *Msx*, and *Prrx* around a core of nested *Dlx* expression along the dorsal–ventral (DV) axis that corresponds to unique structures in the head [16,41]. These genes cooperate with *Hox* genes expressed along the anterior–posterior (AP) axis of the PAs to give rise to transcriptionally distinct CNCC populations in each arch that give rise to skeletal elements of various shapes and properties [16,43]. *Dlx* and *Hox* genes are thought to pattern the DV and AP axes of the vertebrate head in a highly conserved, code-like pattern [16,44].

## 3. Duplicated Genes within the NC GRN

**Figure 2:**
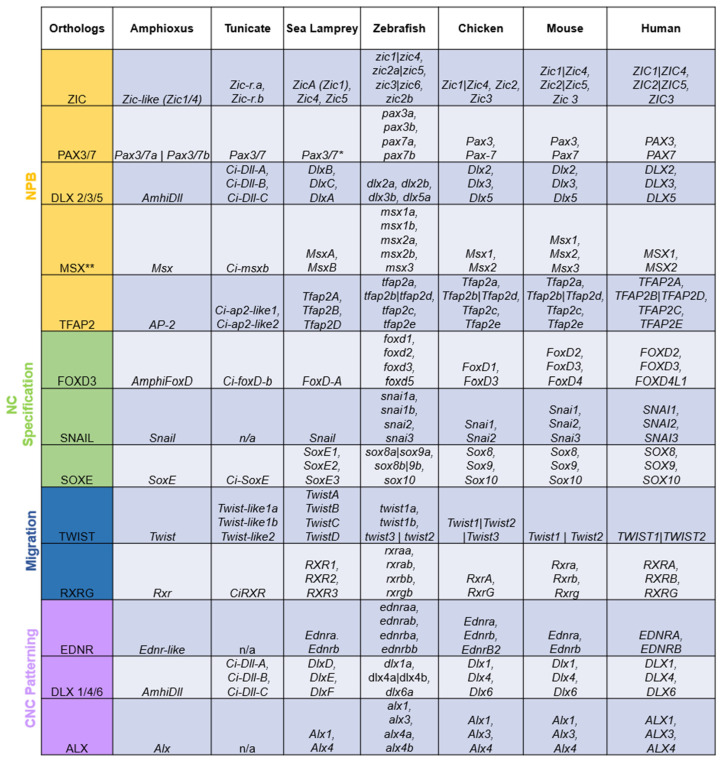
Duplicated orthologs of the NC GRN—amphioxus, tunicate, and sea lamprey orthologs were found in the literature and the NCBI and Stowers Institute databases. Amphioxus [29,46,47,48,49]; tunicate [29,50,51,52,53,54,55,56,57,58,59]; sea lamprey [9,16,23,29,41,60,61,62]. The jawed vertebrate orthologs were collected from the Ohnologs Data Repository [63] and the NCBI gene database. The (|) between gene names indicates that genes are not ohnologs and were duplicated before/after vertebrate-specific WGDs. The (**) on *Msx* orthologs indicates that these genes were marked as pre-2R duplicates according to [64]. The (*) on lamprey *Pax3*/*7* means that there were discrepancies between the annotations of the lamprey genome [65], NCBI, and past work [9].

The term “ohnolog” was coined to distinguish gene duplicates that were products of vertebrate-specific WGDs from paralogs that are exclusive to a single lineage and orthologs shared by multiple lineages [12]. All genes in the NC GRN appear to have been duplicated during the vertebrate WGDs, with the resulting ohnologs being differentially retained across lineages (Figure 2). However, virtually all NC GRN ohnologs function at some point during neural crest development. It would be highly unlikely for each duplicated member of these gene families to be independently co-opted into the same GRN. Thus, the fact that virtually all NC GRN ohnologs are active at some point in the NC GRN, or its various subcircuits, serves as robust evidence that the co-option of these genes to the neural border, and the assembly of the NC GRN, occurred before the WGDs. 

**Figure 3:**
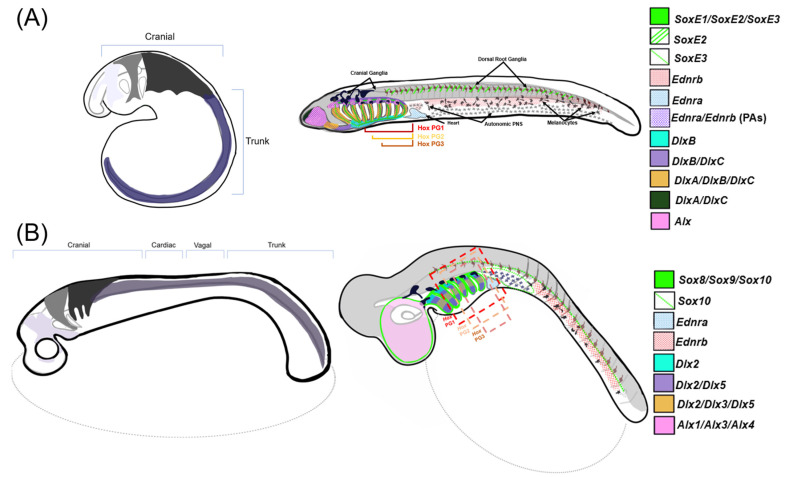
Comparisons of NCC migration and patterning between gnathostomes and lamprey—a stylized diagram of neural crest cell migration and post-migratory gene expression in NCCs within (**A**) sea lamprey and (**B**) a representative gnathostome (catshark). Left: three homologous cranial neural crest streams are displayed migrating into prospective PAs and one generalized trunk stream that migrates posterior of the head. Right: gene expression map of the described NC-GRN orthologs within respective model organisms at the later, pharyngula stage. Note that *Alx1*, *Alx3*, and *Alx4* are expressed differentially along the mediolateral axis in gnathostomes but are depicted as a single expression pattern within the nasopharyngeal region in this diagram [66,67,68].

In this context, the differential expression of NC GRN ohnologs likely reflects the temporal and spatial subfunctionalization of NC GRN components after the WGDs (depicted in Figure 3). The functional consequences of this extensive subfunctionalization of NC GRN ohnologs remain unclear. However, in general terms, these duplications appear to have substantially increased the overall complexity of the modern NC GRN by creating temporally and spatially restricted subcircuits [2,3,7,10,11]. The dedication of individual NC GRN ohnologs to particular phases of the NC GRN, or particular NC subpopulations, may have increased the modularity of the GRN, allowing different portions to evolve without interfering with its other functions. Consider an NC GRN gene that was ancestrally involved in the initial activation of the NC GRN (i.e., NCC specification) and later during the differentiation of two NCC derivations, derivative 1 and derivative 2. Suppose this gene was duplicated during the WGDs, and its three retained ohnologs, A, B, and C, became temporally and spatially subfunctionalized: A being expressed early and dedicated to NCC induction, and B and C expressed later and dedicated to derivative 1 and 2, respectively. If a mutation affected the expression or function of the B ohnolog, causing an adaptive change in derivative 1, it would have minimal effect on NCC specification or the formation of derivative 2. Conceivably, this subfunctionalization of ohnologs could then lead to biochemical or regulatory neofunctionalization, as they evolved new biochemical properties or expression domains. Below, we consider expression and functional data from studies of select duplicated NC genes. We find that this evidence supports the idea that NCCs and the NC GRN evolved before the WGDs. Furthermore, we find support for extensive subfunctionalization followed by some neofunctionalization after the WGDs, suggestive of increased NC GRN modularity [22,23,45].

### 3.1. SoxE

Transcription factors of the *SoxE* family participate in multiple steps of NCC development. While there is one *SoxE* in invertebrate chordates, *SoxE* is duplicated in vertebrates with three each in non-teleost gnathostomes (*Sox8*, *Sox9*, and *Sox10*) and lamprey (*SoxE1*, *SoxE2*, and *SoxE3*) and five in teleosts [4,45,69]. *SoxE3* is in lamprey and gnathostome is in *Sox9*, and both have a conserved role in regulating cartilage morphogenesis. *SoxE2* is comparable to *Sox10*, as both have similar melanogenic and glial-inducing roles [45,69]. All vertebrate *SoxE* paralogs are expressed during induction, migration, and differentiation of NCCs but with heterochronic differences across lineages [22,45,69]. The most parsimonious explanation for this is that *SoxE* was co-opted into the NC GRN before the first WGD at the base of the vertebrates. 1R ohnologs were then duplicated in gnathostomes during the 2R WGD, while one or both 1R ohnologs were independently duplicated in the cyclostome lineage. Based on the similar expression of *SoxE3*/*Sox9* in CNCCs and *SoxE2*/Sox10 in the trunk, the subfunctionalization of R1 *SoxE* duplicates likely occurred before the gnathostome/agnathan split. 

Interestingly, *AmphiSoxE* is expressed in the fibrillar cartilage of the oral cirri in amphioxus, pointing to an ancestral role for *SoxE* in cartilage development [4]. *AmphiSoxE* is capable of activating downstream genes in NC GRN modules, such as neurogenic *Phox2* and melanogenic *Mitf*, within transgenic zebrafish, expressing *AmphiSoxE* and other NC specifiers in transgenic chicks [22,45]. This indicates an ancestral function in differentiation and NCC induction in the ancestral *SoxE*. However, *AmphiSoxE* regulatory elements are not capable of driving reporter expression within NCCs in transgenic zebrafish, indicating that a *cis* change permitted *SoxE* function in neural crest GRN [4]. Additionally, transgenic mice expressing the *Drosophila* ortholog of *SoxE* (*Sox100B*) in place of *Sox10* developed many NC derivatives, including the peripheral nervous system (PNS) and melanocytes, showing that *Sox10* has retained a highly conserved role of inductive capabilities [70]. However, when *Sox8* is expressed in place of *Sox10*, transgenic mice fail to develop melanocytes, indicating that gnathostome *Sox8*/*9*/*10* proteins have diverged in functionality [3,71]. Taken together, these data suggest that after an ancestral *SoxE* was co-opted into the NC GRN by a *cis*-regulatory mutation, it underwent duplication and both temporal and spatial subfunctionalization of its ancestral expression pattern. This was followed by some divergence of the *SoxE* protein function, reflecting either subfunctionalization or neofunctionalization of *SoxE* ohnologs.

### 3.2. Dlx 

The Dlx transcription factor family, which underwent an ancient tandem duplication in the last common ancestor of vertebrates and urochordates, is homologous to the *distal-less* gene (*Dll*) in insects [72]. This tandem gene pair was duplicated in gnathostomes into pairs of multiple copies across different chromosomes in a pattern that agrees with 2R duplication events [72,73]. As with other gene families, *Dlx* orthology is not perfect between cyclostomes and gnathostomes, and teleosts have more *Dlx* paralogs than other gnathostomes. Each tandem duplicate has its own orthology group, annotated *Dlx1*/*4*/*6* and *Dlx2*/*3*/*5*, in gnathostomes. While in lamprey, *Dlx* orthologs are annotated as *DlxD*/*E*/*F* and *DlxA*/*B*/*C*, respectively [16,73]. The last common vertebrate ancestor is hypothesized to have duplicated its tandem *Dlx* clusters during the 1R event with lineage-specific duplications followed by differential retention, resulting in all vertebrates having six *Dlx* genes [16,72,73]. 

The *Dlx* family has a highly conserved role in patterning, especially within the vertebrate head where it exhibits a nested expression pattern along the DV axis of the Pas; however, the precise borders of these expression patterns vary between jawed and jawless vertebrates [16,74]. The sea lamprey exhibits nested DV expression of *DlxA-D* paralogs and regionalized *Hand1* and *Msx* transcription, reminiscent of the *Dlx-Hand-Msx* code in gnathostomes [75]. This nesting is less obvious in the Japanese lamprey with the exception of *DlxF*, which is differentially expressed in the nasal region [43]. As in gnathostomes, the combinatorial differences in *Dlx*, *Hand*, and *Msx* expression domains along the DV axis also correlate with different cartilage types within the lamprey head [75].

As with other NC GRN genes, all *Dlx* paralogs are expressed in NCC at some point during NCC development, strongly supporting the idea that the ancestral *Dlx* gene pair was co-opted into the NC GRN before the WGDs [16]. The distinct expression patterns of *Dlx* ohnologs further suggest the subfunctionalization of an ancestral, pan-NCC expression pattern. The fact that different combinations of *Dlx* genes mark different portions of the head skeleton is consistent with the participation of different *Dlx* ohnologs in separate regulatory subcircuits in the NC GRN. The degree to which the biochemical function of the transcription factors encoded *Dlx* genes was affected by WGDs is unclear, as there is limited evidence that *Dlx* proteins have qualitatively different DNA binding properties. However, the divergent expression of *Dlx* ohnologs supports the idea that the WGDs allowed the regulatory landscape of the *Dlx* family to diverge, resulting in the nested expression of the “*Dlx* Code” [40]. The complex expression of *Dlx* ohnologs within the vertebrate head may be consistent with the idea that the patterning of NCC derivative became more elaborate following the vertebrate-specific expansion of this gene family.

### 3.3. Hox Clusters

The *Hox* gene family has been presented as evidence for the 2R hypothesis, one reason being that amphioxus possesses a single, syntenic cluster of tandem *Hox* homologs, while gnathostomes have four homologous clusters across separate chromosomes [76,77,78]. *Hox* clusters in teleosts are also evidence for teleost-specific genome duplication (3R) and are distributed across seven chromosomes [72]. Alternatively, some have used data from phylogenetic analyses of human *Hox* genes as evidence against WGDs, and propose that this gene family was expanded by small-scale events that occurred in vertebrates [79,80,81]. There are ancient paralogical groups of *Hox* genes that are annotated numerically within clusters (i.e., *Hox1*, *Hox2*, *Hox3*…). These groups are *cis-*tandem duplicates that occur throughout metazoans, and *Hox* ohnologs are specified by letters (i.e., *Hoxa1*, *Hoxb1*, *Hoxc1*…) and occupy separate chromosomes in vertebrates. These transcription factor genes are expressed in a conserved, colinear manner across bilaterians along the anterior–posterior axis and have been shown to induce homeotic transformations if mutated [82,83,84,85].

Within the pharyngeal arches of all vertebrates, the first PA (PA1) is *Hox-*negative, PA2 is marked by *Hox2* paralogs, and PA3, along with the posterior arches, are marked by *Hox3* paralogs [43,84,86,87]. The ectopic expression of *Hox cis*-paralogs within the PAs can lead to homeotic transformations, for instance when *Hoxa2* is expressed in the first PA of mice, which is *Hox-*negative, embryos lose the mandibular structures or take on a second arch identity [88]. Additionally, certain levels of ectopic *Hoxa2* expression can alter skeletal element identity in mice, highlighting the possibility that the regulation of Hox gene expression could have more of an influence on skeletal identity than protein function [88]. Knock-downs of *hox2* ohnologs in zebrafish have shown that these genes are partially redundant, as removing the function of one ohnolog often results in a less dramatic phenotypic effect than targeting the whole group [85,89]. This highlights the functional differences between Hox tandem duplicates, yet the degree to which Hox ohnologs differ regarding DNA binding specificity is still unclear.

In cyclostomes, the *Hox* gene orthology is disparate from gnathostomes, providing another line of evidence for cyclostome-specific duplications subsequent to their divergence from jawed vertebrates and the possibility of segmental gene duplications in this family [44,65]. Differences in the *Hox* cluster number and organization between different species of lamprey and hagfish add difficulty to solving the phylogeny of *Hox* clusters [44,84]. Although *Hox* orthologues are hard to assign between jawed and jawless vertebrates, there is evidence that *cis-*regulatory elements (CREs) upstream of *Hoxa1*/*Hoxb1* in gnathostomes and *hoxα1* in lamprey are capable of driving similar expression in NC [44]. Similar CRE activity provides minor support for orthology assignment as well as the nested colinear expression pattern of tandem duplicates in the pharyngeal arches [44,84]. *Hox* ohnologs have been shown to have both redundant and diverged traits in NC patterning, making them a complex gene family for understanding their evolution after duplication events. Recent work in zebrafish has shown that within the PAs, certain levels of ectopic *hoxa2* expression can alter skeletal element identity, highlighting the possibility that the regulation of *Hox* gene expression could have more of an influence on skeletal identity than protein function [88]. More transgenic experiments between various gnathostomes and cyclostomes of Hox ohnologs would highlight whether vertebrate WGDs allowed this gene family to diversify. Isolating protein-coding regions and CREs within the experiments would expand our current understanding of how or whether WGDs impact gene evolution. Specifically, phenotypes seen within the PAs following these gene swap experiments would highlight how duplication events shaped the patterning of NCCs in the head and whether these gene expansions were necessary for the new head to evolve.

### 3.4. EdnR

Cranial neural crest cells have endothelin receptors that interpret signals from the surrounding tissues to activate downstream genes, including NCC specifier genes and *Dlx* [16,23,41]. The endothelin receptor genes (*Ednra*/*Ednrb*) are both critical components of the NC GRN, suggesting they are likely “1R” duplicates of a single ancestral *Ednr* present in the last common vertebrate ancestor [23,90]. The 2R WGD in gnathostomes was presumably followed by the loss of two *Ednrs*, leaving only one *Ednra* and one *Ednrb*, while the teleost 3R resulted in four *Ednr* genes in this group. Following the gnathostome–cyclostome split, *Ednrs* evolved somewhat divergent roles in CNCC differentiation [23,91]. Within gnathostomes, *Ednra* has a key role in skeletogenesis and vascular development, while *Ednrb* contributes to melanocyte and peripheral nervous system development, and *Ednrb* mutants have no craniofacial defects [92]. In contrast, lamprey *Ednra* and *Ednrb* both are required for proper PA formation, and those lacking either *Ednra* or *Ednrb* fail to form branchial arch cartilages properly [23,92]. Recently, it was discovered that skate embryos also express *Ednrb* in the ventral and intermediate NCCs in all pharyngeal arches in a way that resembles lamprey expression [93]. Together, these data imply that after the R2 WGD, *Ednr* ohnolog expression diverged in chondrichthyans and bony fish, with the developmental roles of *Ednra* and *Ednrb* becoming more specialized in the latter [23,94].

Endothelin receptors on the surface of NCCs activate genes that contribute to the skeletal phenotype within each arch. Within gnathostomes, *Ednra* activates *Hand* and *Dlx5*/*6* genes within the ventral portion of the PAs in zebrafish, mice, and frogs, where they contribute to the lower jaw and joint formation [23,92,95]. Lamprey exhibit similar *Dlx* expression within the ventral Pas; however, hand expression is not regulated by *Ednra* [23]. Duplicated genes can subfunctionalize or neofunctionalize following duplications; *Ednra* and *Ednrb* appear to have diverged in terms of expression pattern and downstream gene targets [23,41]. It is possible that a stem or independent duplication provided the flexibility necessary for agnathans and gnathostomes to alter endothelin signaling, supporting the morphological specialization of their head skeletons. Gene swap experiments between cyclostome and gnathostome *Ednra*/*Ednrb* orthologues will highlight the divergent aspects of these paralogous signaling pathways. *Ednra*/*Ednrb* divergence following duplication is a potential link between head specialization and “2nd R”, but further research will have to be conducted to deem it as a necessary event for that divergence.

### 3.5. Alx

*Alx* genes are part of NC GRN subcircuits that confer cellular identity in NCC-derived skeletal elements, contributing to their unique shapes and different components [16,41,66]. This gene family has a conserved skeletal role across deuterostomes and is deployed in the skeletal GRNs of echinoderms and vertebrates [96]. Amphioxus possesses two copies of *Alx* (*Bf-alx1*, *Bf-alx2*)m whose loci are near each other and share highly similar intron/exon structures, indicating a lineage-specific, tandem duplication [96]. Non-teleost gnathostomes have three *Alx* homologs, *Alx1*/*Cart1*, *Alx3*, and *Alx4*, and teleosts possess additional paralogs of *Alx4* (*alx4a*, *alx4b*) that are generated during the vertebrate and teleost WGDs, demonstrating that these paralogs have been differentially retained [66,96]. Lamprey possesses two *Alx* homologs that were likely generated during the first vertebrate WGD and orthologous to gnathostomes *Alx1* and *Alx4* [41]. Only the expression of lamprey *Alx4* has been reported, and like gnathostome *Alx* ohnologs, it is expressed in skeletogenic CNCCs [41].

The expression of different *Alx* paralogs corresponds to different cellular cartilage shapes within the vertebrate head [16,66,67,75,96]. In lamprey, *Alx4* expression is largely coincident with stiffer, more rigid cartilage phenotypes, indicating an ancestral role in the differentiation of skeletal tissue subtypes [41]. In zebrafish, combinations of *Alx* paralogs label NCC-derived chondrocytes with distinct cellular phenotypes. Detailed functional analyses suggest that this “*Alx* code” contributes to the intricate shapes of the gnathostome head skeleton [66]. In mice, *Alx1* is activated earlier and is concentrated in the midline regions of the frontal nasal prominence (FNP), while *Alx3* and *Alx4* are largely limited to the lateral tissues of the FNP [67,68]. Experiments in mice have also shown that *Alx* paralogs have diverged in function, with homozygous deletions of *Alx1* or *Alx3* resulting in different craniofacial malformations, while *Alx4* loss-of-function results in minimal defects [67]. Deciphering the evolution of the gnathostome “*Alx* code” would be aided by gene swap experiments within and between species or the overexpression experiments of various Alx1/3/4 paralogs. Furthermore, analyzing the open chromatin regions surrounding paralogs within various lineages will help determine whether regulatory changes are at the base of their divergence or if there are protein sequences that have diverged biochemically. 

## 4. Conclusions and Future Directions

The evolution of bona fide NCCs is central to the new head hypothesis. At the genomic level, the evolution of truly novel cell types requires the evolution of new GRNs, and several scenarios have been proposed for the initial assembly of the NC GRN. These include the stepwise acquisition of regulatory interactions between individual genes, the co-option of small genetic subcircuits from other germ layers, and the heterochronic reactivation of whole GRNs [4,5,7,10,25,35]. None of these mechanisms are mutually exclusive with each other or the 2R hypothesis. The degree to which the vertebrate WGDs were necessary for the origin and/or subsequent evolution of the modern NC GRN and NCCs is unclear. Here, we assess genomic structure, gene expression, and functional data from a range of vertebrates and invertebrate deuterostomes to better understand the role of the vertebrate WGDs in the evolution of NCCs and the new head. The observations that (1) all core NC GRN components have invertebrate homologs and (2) virtually all paralogs of these genes function in the NC GRN strongly support the idea the NC GRN (and thus NCC cells) first evolved in a vertebrate ancestor with an unduplicated genome. Thus, the WGDs were likely not the main factor driving the origin of the vertebrate new head.

However, the timing of the vertebrate WGDs does appear to correlate with three key radiation events, each of which was characterized by extensive diversification of the head skeleton. The timing of the first “1R” WGD coincided with the initial radiation of agnathan vertebrates when the lineages leading to modern cyclostomes, gnathostomes, and extinct forms, like galeaspids and osteostracans, diverged [15]. The second “2R” duplication was likely gnathostome-specific and preceded the massive radiation that ultimately led to this group’s ecological dominance [2,23]. Finally, the “3R” WGD duplication in the teleost lineage corresponds to taxon-defining skeletal innovations in this group through the explosive diversification of teleosts, which likely occurred 50–100 million years after 3R [14]. In addition to coinciding with the repeated diversification of the new head, the WGDs also appear to have driven an increase in the complexity of the NC GRN. Specifically, the temporal and spatial subfunctionalization of duplicated NC GRN genes, including *SoxE*, *Ednr*, *Dlx*, and *Alx*, facilitated the evolution of new NC GRN subcircuits restricted to different NCC subpopulations. Thus, after the WGDs, new subcircuits evolved that were dedicated to cranial versus trunk NCCs, skeletogenic versus non-skeletogenic NCCs, dorsal versus ventral cranial NCCs, medial versus later cranial NCCs, etc. We speculate this proliferation of spatiotemporally restricted NC GRN subcircuits facilitated the diversification of CNCC derivatives by allowing different NCC subpopulations to evolve independently.

In sum, while it is likely WGDs were not necessary for the initial origin of NCCs and the new head, several lines of evidence point to a role for the WGDs in the evolutionary diversification of the vertebrate head. A testable prediction of this hypothesis is that subpopulation-specific NC GRN subcircuits have diverged in different vertebrate lineages, leading to morphological differences in NCC-derived structures. In the simplest terms, the evolutionary divergence of an NC GRN subcircuit could result from mutations in the *cis*-regulatory and/or protein-coding portions of NC GRN genes. Several studies support the divergent functions of duplicated NC GRN genes, most of which encode transcription factors [23,66,68,71]. However, this work largely involves loss-of-function experiments, showing that paralogous genes regulate different target genes. These experiments typically do not distinguish between the differential regulation of target genes due to mutations in CREs versus differences in DNA binding abilities in paralogous transcription factors. A deeper and more nuanced understanding of the extent and morphological impact of NC GRN paralog divergence could be achieved by swapping paralogous protein-coding sequences, as has been conducted with *SoxE* family members in mice (see the “*SoxE*” section above). For instance, swapping paralogs within and between vertebrate species would test if *Dlx* and *Alx* protein function underlie differences in skeletal element morphology, as predicted by the “*Dlx* code” and “*Alx* code” hypotheses. Similar analysis of the CREs associated with NC GRN genes could reveal the degree to which the evolution of new CREs permitted the diversification of NC derivatives, like the head skeleton. In addition to shedding light on vertebrate evolution, such comparative experiments would also illustrate some of the general principles by which gene duplication facilitates the evolution of new GRNs, cell types, and morphologies.

## Data Availability

Not applicable.
